# Trends in Second‐Trimester Safe Abortion Service Utilization During the Two Phases of COVID‐19 Pandemic in an Ethiopian Setting: A Cross‐Sectional Study

**DOI:** 10.1002/puh2.210

**Published:** 2024-06-28

**Authors:** Abraham Fessehaye Sium, Don Eliseo Lucero‐Prisno

**Affiliations:** ^1^ Department of Obstetrics and Gynecology St. Paul's Hospital Millennium Medical College (SPHMMC) Addis Ababa Ethiopia; ^2^ Faculty of Management and Development Studies University of the Philippines (Open University) Los Baños Laguna Philippines

**Keywords:** COVID‐19, MICHU clinic, pandemic, second‐trimester abortion, surgical abortion

## Abstract

**Background:**

Providing abortion services during pandemics without interruption is essential. The objective of the study was to compare the utilization of second‐trimester safe abortion care services between early pandemic and peak pandemic months.

**Methods:**

We conducted a retrospective comparative study of second‐trimester safe abortion service provision during the peak of COVID‐19 pandemic in Ethiopia (April 2021) versus during that of early pandemic (April 2020). Data were collected using data extraction form from MICHU clinic HMIS registry. Data were analyzed using SPSS version 23. Chi‐squared test and simple descriptive statistics were applied as appropriate. A *p*‐value of less than 0.05 was used to describe the significance of the results.

**Results:**

There were significantly more second‐trimester abortions performed during the peak pandemic month as compared to early pandemic month (32.6% vs. 67.4%, respectively). Nearly half of the abortions were done for maternal health problems (47.8%, 44/92), and of which over 80% of them used medical methods for abortion (83.7%, 77/92). There was only one case of dilation and evacuation (D&E) during the early pandemic, compared to 14 D&E procedures during the pandemic peak.

**Conclusion:**

We found a significant increment in the second‐trimester abortion service coverage during the peak of COVID‐19 pandemic. The measures we took during the early pandemic: increasing public awareness about continuation of abortion care services during the pandemic, change in the attitude of care‐providers, and early sticking to national guidelines on essential care during COVID‐19 should be passed on as important lessons for future pandemics.

## Graphical abstract

Providing abortion services during pandemics without interruption is essential. We conducted a retrospective comparative study, and we found a significant increment (an increase by 107%, *p*‐value = 0.019) in the second‐trimester abortion service coverage during the peak of COVID‐19 pandemic (April 2021) compared to during early COVID‐19 pandemic (April 2020).

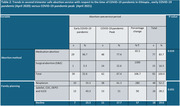

## 1 Introduction

Induced abortion is a common reproductive health experience, each year about 56 million induced abortions occur globally, of which nearly 7 million women in developing countries develop complications from unsafe abortions. An estimated 22,000 women die from abortion‐related complications annually [1]. Abortion is time‐ sensitive, and attention should be paid to providing care as early as possible given gestational limits [2]. There is evidence that abortion rates are similar whether access to abortion is freely available or restricted, but that where access is restricted, women are more likely to resort to unsafe abortion outside of medical regulation, which is likely to be detrimental to both them and the healthcare system [3]. In fact, there are 25 million unsafe abortions per year, 30 women die for every 100,000 unsafe abortions in developed regions and 220 deaths per 100,000 unsafe abortions in developing regions [4]. Second‐trimester medical abortion is termination of pregnancy between 12 and 28 weeks of gestational age [5]. Second‐trimester abortions constitute 10%–15% of all induced abortions worldwide but are responsible for two‐thirds of major abortion‐related complications [6].

Abortion services were more essential during the COVID‐19 pandemic than in any of the past pandemics. Reports indicated that COVID‐19 responses lead to increased unintended and unwanted pregnancies due to quickly diminishing contraceptive supplies, increased incidence of domestic violence, and rising income insecurity. Compelling continuation of unwanted pregnancies is recognized as a human rights violation in several circumstances, including where there are foreseeable physical or mental health impacts for pregnant persons [7]. Despite abortion care being necessary to ensure the health and safety of many women, the COVID‐19 pandemic has exacerbated existing disparities in access that exist in many countries globally. In response to the impact of the circumstance of the pandemic, providing early medical abortion through telemedical services was recommended [8]. However, delayed abortion care in the second trimester cannot be provided with those innovative methods because it requires physician–patient contact and it is given as inpatient care, provided that it contributes to two‐thirds of abortion‐related major complications. Second‐trimester abortion provides women with an intended pregnancy the last chance of accessing abortion care before abortion denial ensues due to upper gestational age limit (especially those that present in the late second trimester at ≥20 weeks), and the women are forced to carry the pregnancy to delivery, take the burden of raising unwanted children, in countries where abortion law permits second‐trimester abortion up to 28 weeks. Hence, it is always essential not to lose this last opportunity of having abortion for women who already present late for different reasons, apart from inaccessibility of care on time due to pandemics. It was imperative that second‐trimester abortion care remains uninterrupted (including that in the late second trimester) during the pandemic. This study aimed to determine the trends in second‐trimester safe abortion care in an Ethiopian setting during the two phases of COVID‐19 pandemic (early pandemic vs. peak of the COVID‐19 pandemic).

## 2 Methods

### 2.1 Study Design, Area, and Period

This study is a retrospective cross‐sectional study of second‐trimester safe abortion service utilization during the peak of COVID‐19 pandemic in Ethiopia (April 2021) compared to during early pandemic (April 2020). It was conducted in St. Paul's Hospital Millennium Medical College (SPHMMC) at MICHU clinic, in Addis Ababa, Ethiopia. MICHU clinics are organized family planning and comprehensive abortion care (CAC) clinics that deliver comprehensive service across many regions of Ethiopia. The mother MICHU clinic is a center of excellence for family planning and CAC, and it is located at SPHMMC. Family planning fellows, family planning subspecialists, residents, and midlevel abortion care‐providers are the care‐providers at this clinic. The clinic delivers abortion service to one of the highest numbers of clients in the country—it provides both first‐ and second‐trimester abortion services (up to the gestational age of viability—which is 28 weeks in Ethiopia).

**FIGURE 1:**
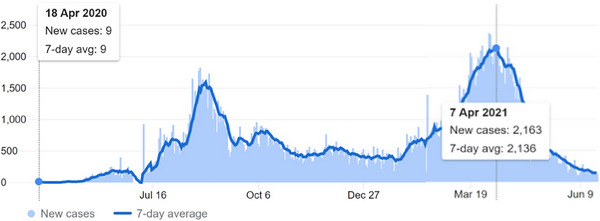
JHU CSSE COVID‐19 data for Ethiopia updated on June 20, 2021 [9].

We retrospectively analyzed the data for second‐trimester (12–28weeks) abortion cases over 2 months (April 2020—during early pandemic and during pandemic peak—April 2021). We defined early COVID‐19 pandemic (April 2020) as the month in which the daily record of cases was the lowest; there were less than 50 cases of COVID‐19 recorded in Ethiopia during this month, whereas there were 49,670 COVID‐19 cases recorded in the month of April 2021 with the highest daily record of 2163 cases of COVID‐19, which we labeled pandemic peak, which is one of the highest (Figure 1).

### 2.2 Sample Size and Sampling Procedure

No sample size calculation applied (all cases of second‐trimester safe abortion cases who received care at MICHU clinic at the mentioned period of time were included based on inclusion and exclusion criteria were included in the study).

#### 2.2.1 Inclusion Criteria

1. Safe abortion care—both medication abortion and surgical abortion
2. Second‐trimester pregnancy (12–28 weeks)
3. Safe abortion care provided started at MICHU Clinic and completed at procedure room.

#### 2.2.2 Exclusion Criteria

1. Postabortion care
2. Molar pregnancy

### 2.3 Data Collection and Variables

Data were extracted from HMIS registry book at MICHU clinic and procedure room (Part of MICHU clinic where clients receive in‐patient care) registry. We used a structured data extraction form written in English to extract the data. Data for the following variables were collected; maternal age; gestational age at termination, indication for safe abortion, method of abortion, family planning provided, and abortion complications. All filled questionnaires were checked for completeness, accuracy, and consistency by the principal investigator. Supervision was carried out by the principal investigator throughout the data collection.

### 2.4 Data Analysis

Data were analyzed using SPSS version 23. Simple descriptive statistics were used to describe socio‐demographic characteristics and abortion service provision during both months April 2020 and April 2021. Chi‐ squared test was applied to test the association of timing of the pandemic with second‐trimester abortion service utilization, and *p*‐value less than 0.05 was used to describe the findings significance.

### 2.5 Ethical Considerations

Ethical clearance was obtained from the Institutional Review Board (IRB) of SPHMMC. The requirement for obtaining informed consent from the study participants was waived by this ethics committee. Data anonymity and confidentiality were maintained throughout the study.

## 3 Results

A total of 92 cases of second‐trimester abortion cases were retrieved from the HMIS, out of which 30 cases received care in April 2020 (Early pandemic) and the rest 62 cases were managed in April 2021 (pandemic peak). The median maternal age and the gestational age were comparable between early pandemic and peak pandemic months. Of 92 study population, 75% (69/92) were primigravida. There were significantly more second‐trimester abortions performed during the peak pandemic months as compared to early pandemic months (32.6% vs. 67.4% for early pandemic and peak pandemic months, respectively). Nearly half of the second‐trimester abortions were performed due for maternal health reasons, and of which, over 80% of them used medical methods of abortion (83.7%, 77/92). Nexplanon was the most preferred family planning method during the peak pandemic month as compared to early pandemic (33.3% vs. 61.3%, respectively). The details are shown in Table 1.

**TABLE 1:** Baseline characteristics of second‐trimester abortion cases managed during the pandemic in Ethiopia, April 2020 (early COVID‐19 pandemic) versus April 2021 (COVID‐19 pandemic peak).

		Abortion care service period							
		Early COVID‐19 pandemic		COVID‐19 pandemic Peak		Percentage change	Total		
Characteristics	Category	*n*	%	*n*	%	%↑	*n*	%	*p*‐Value
Gravidity	Primigravida	21	70.0	48	77.4	129	69	75.0	0.441
	Multigravida	9	30.0	14	22.6	55.6	23	25.0	
Abortion method	Medication abortion	29	96.7	48	77.4	65.5	77	83.7	0.019
	Dilation and evacuation	1	3.3	14	22.6	1300	15	16.3	
Indication for safe abortion	Rape and incest	3	10	26	41.9	1150	29	31.5	0.007
	Maternal health	20	66.7	24	38.7	20	44	47.8	
	Fetal congenital anomaly	7	23.3	12	19.4	71	19	20.7	
Gestational age	Median	20		20			20		1.00
Maternal age	Median	25		21			22		1.00

### 3.1 Trends in Second‐Trimester Safe Abortion Service With Respect to Time of COVID‐19 Pandemic

Among significant findings of this study, a high jump in the number of second‐trimester safe abortion cases during the pandemic peak (April 2021) was observed (Table 2). A record 107% (*p*‐value = 0.019) increase was seen during the pandemic peak (62 cases vs. 30 cases) in comparison with the second‐trimester abortion care during the early pandemic.

**TABLE 2:** Trends in second‐trimester safe abortion service with respect to the time of COVID‐19 pandemic in Ethiopia, early COVID‐19 pandemic (April 2020) versus COVID‐19 pandemic peak (April 2021).

		Abortion care service period							
		Early COVID‐19 pandemic		COVID‐19 pandemic peak		Percentage change	Total		
Variable		*N*	%	*n*	%	%↑	*n*	%	*p*‐Value
Abortion method	Medication abortion	29	96.7	48	77.4	65.5	77	83.7	0.019
	Surgical abortion (D&E)	1	3.3	14	22.6	1300	15	16.3	
	Total	30	32.6	62	67.4	106.7	92	100.0	
Family planning	Nexplanon	10	33.3	38	61.3	280	48	52.2	0.031
	Jadellel, COC, DEPO, and IUCD	13	43.3	13	21	50	26	28.2	
	Declined	7	23.3	11	17.7	57	18	19.6	

Abbreviation: COC, combined oral contraceptives; D&E, dilation and evacuation; DEPO, Depo‐Provera; IUCD, intra‐uterine contraceptive device

A similar high jump was also observed in abortion methods utilized (Table 2) during the peak pandemic (April 2021) compared to the early pandemic (April 2020). A record of 1300% (*p*‐value = 0.019) increase in surgical abortion cases was observed. There was only one case of dilation and evacuation (D&E) during the early pandemic, which is much lower than the 14 D&E procedures during the pandemic peak (*p*‐value = 0.019).

## 4 Discussion

In this study, we found that the second‐trimester abortion service delivery was higher during the peak of COVID‐19 pandemic than during the early COVID‐19 pandemic. Second‐trimester abortion cases increased by more than 10‐fold during the pandemic peak in April 2021 compared to the early pandemic (April 2020).

Because of the COVID‐19 pandemic, many problems have emerged in the organization of the National Health Systems [10]. Both government and private setups in most parts of the world suspended many routine and elective services. There were some essential health services, which by no means should suffer because of the COVID‐19 pandemic, and abortion care services are one of them [11]. In Ethiopia, as a result of the preventive measures by the government and fear of risk of exposure by the community, it was anticipated that utilization of reproductive, maternal, newborn, and child health services might be affected, and huge efforts were made in response to the pandemic, not to leave sexual and reproductive health services behind. In our study, there was a sharp increase in the number of second‐trimester safe abortion cases during the pandemic peak, compared to the early COVID‐19 pandemic. In line with this, another study done in the same setting where our study was conducted found a negative effect of COVID‐19 on family planning and safe abortion care service provision during the early pandemic [12]. The decrement in sexual and reproductive health service utilization, including abortion care, found in this previous study was attributed to the impact COVID‐19 pandemic in terms of negative care‐provider, clients fear of contracting COVID‐19 at health facility, transportation shortage, and misconceptions that health facilities were not providing essential health care services. In addition to the huge effort made at the ministry level (in Ethiopia) not to leave sexual and reproductive health behind during the COVID‐19 pandemic, public awareness creation through media in a form of interview with physicians, uninterrupted functioning of our abortion care services, proper triaging and screening for COVID‐19, and early addressing of negative attitude of care‐providers were among the efforts made at our hospital following understanding of these gaps from that earlier study. This probably explains the reason why this surge in the number of second‐trimester cases was found compared to the early pandemic, which is contrary to the fact that in the rest of the world, health services, including sexual and reproductive, were hampered badly by the pandemic [13].

In our study, a 107% increase in the number of second‐trimester abortion cases occurred during the pandemic peak (62 cases vs. 30 cases) in comparison to that of during the early pandemic. A high increase was also observed in the surgical abortion cases during the peak pandemic (April 2021) compared to the early pandemic (April 2020). More than 10‐fold increase was observed in the number of surgical abortion cases during the pandemic peak. There was only one case of D&E during the early pandemic, which was much lower than the 14 D&E cases during the pandemic peak. These results show that a higher negative impact of the panic phase (early phase) of the pandemic on second‐trimester abortion care was experienced than the pandemic peak (when one may expect severe compromisation of abortion service delivery).

Findings of increased second‐trimester abortion service delivery during the peak of COVID‐19 pandemic in our study, compared to the early pandemic (April 2020), have several practice implications for future pandemics. Importantly, it implies that the panic phases of pandemics have higher negative impact on second‐trimester abortion service delivery than the reality (facing the peak of the pandemics). By changing attitude of care‐provider on service provision during COVID‐19 pandemic, COVID‐19 public awareness creation through volunteered experts’ interviews on media and a rapid implementation of guidelines of the Ministry of Health of Ethiopia on essential health services, including sexual and reproductive health, were the main measures taken to improve service provision during the early pandemic in our setting. These measures could be adopted for future pandemics when it comes to second‐trimester service continuation amid future pandemics.

Strengths of this study include the ability to compare the impact of early pandemic on second‐trimester abortion to that of the pandemic peak in an Ethiopian setting, respective month to month comparison‐lessening cyclic effect on service provision. The second strength of the study is the ability to put the results into Ethiopian perspective of the pandemic. A graphic description of the COVID‐19 cases in Ethiopia during early pandemic and peak of the pandemic in relation to the trends of safe second‐trimester abortion is an additional strength of the study. Limitations of the study include the nature of the study which is retrospective study and the inability to control the other potential confounders that may have contributed to these results. Adding a qualitative information from patients and care‐providers could have enriched the conclusion of this study, by determining other associated factors that could have played a role in reversing the course of safe second‐trimester abortion care in our study setting. Not including data analysis in the trends of second‐trimester abortion before the pandemic (the same month April 2019) is the other limitation of our study.

## 5 Conclusion

In this study, we found significant improvement in the second‐trimester abortion delivery services during the peak of COVID‐19 pandemic compared to that during early pandemic. There was more than 100% increase in second‐trimester abortion service delivery during the pandemic peak. Public awareness creation on the importance of not missing essential care during the pandemic and change in the attitude of care‐providers were some of the measures taken during the early pandemic. Parallel to this, we sticked early to Ministry of Health guidelines on essential care during COVID‐19. These measures probably have resulted in such improvement in second‐trimester abortion care delivery during the COVID‐19 pandemic peak. These measures in the light of our study findings should be passed on as important lessons for future pandemics.

## Author Contributions

A.F.S. developed the concept and design of the article and data collection. A.F.S. and D.E.L. analyzed the data. Both authors critically revised the article for intellectual content and gave final approval for submission for publication.

## Ethics Statement

Ethical clearance was obtained from the Institutional Review Board (IRB) of St. Paul's Hospital Millennium Medical College (SPHMMC). The requirement for obtaining informed consent from the study participants was waived by this ethics committee.

## Conflicts of Interest

The author declares no conflicts of interest. Abraham Fessehaye Sium is an Editorial Board member of Public Health Challenges and co‐author of this article. Don Eliseo III Lucero‐Prisno is the Editor‐in‐Chief of Public Health Challenges and co‐author of this article. They were excluded from editorial decision‐making related to the acceptance of this article for publication in the journal.

## Data Availability

The datasets generated and analyzed during the current study included in the final version submitted for publication.
